# EVs-miR-17-5p attenuates the osteogenic differentiation of vascular smooth muscle cells potentially via inhibition of TGF-β signaling under high glucose conditions

**DOI:** 10.1038/s41598-024-67006-9

**Published:** 2024-07-15

**Authors:** Isashi Baba, Tetsuya Matoba, Shunsuke Katsuki, Jun-ichiro Koga, Takuro Kawahara, Mitsukuni Kimura, Hidetaka Akita, Hiroyuki Tsutsui

**Affiliations:** 1 Department of Cardiovascular Medicine, Graduate School of Medical Sciences, Kyushu University, 3-1-1, Maidashi, Higashi-ku, Fukuoka, 812-8582 Japan; 2 Second Department of Internal Medicine, School of Medicine, University of Occupational and Environmental Health, Kitakyushu, Japan; 3 Laboratory of Drug Design and Drug Disposition, Graduate School of Pharmaceutical Sciences, Tohoku University, Sendai, Japan

**Keywords:** Calcification, miRNAs, Extracellular signalling molecules

## Abstract

Vascular calcification, which is a major complication of diabetes mellitus, is an independent risk factor for cardiovascular disease. Osteogenic differentiation of vascular smooth muscle cells (VSMCs) is one of the key mechanisms underlying vascular calcification. Emerging evidence suggests that macrophage-derived extracellular vesicles (EVs) may be involved in calcification within atherosclerotic plaques in patients with diabetes mellitus. However, the role of macrophage-derived EVs in the progression of vascular calcification is largely unknown. In this study, we investigated whether macrophage-derived EVs contribute to the osteogenic differentiation of VSMCs under high glucose conditions. We isolated EVs that were secreted by murine peritoneal macrophages under normal glucose (EVs-NG) or high glucose (EVs-HG) conditions. miRNA array analysis in EVs from murine macrophages showed that miR-17-5p was significantly increased in EVs-HG compared with EVs-NG. Prediction analysis with miRbase identified transforming growth factor β receptor type II (TGF-β RII) as a potential target of miR-17-5p. EVs-HG as well as miR-17-5p overexpression with lipid nanoparticles inhibited the gene expression of Runx2, and TGF-β RII. Furthermore, we demonstrated that VSMCs transfected with miR-17-5p mimic inhibited calcium deposition. Our findings reveal a novel role of macrophage-derived EVs in the negative regulation of osteogenic differentiation in VSMCs under high glucose conditions.

## Introduction

Vascular calcification is a life-threatening risk factor for cardiovascular diseases, and it is a well-known complication that occurs in patients with chronic kidney disease, diabetes mellitus, and atherosclerosis^1^. The severity of vascular calcification depends on glucose intolerance, especially in patients with diabetes mellitus^2^, and the mortality and morbidity of atherosclerotic cardiovascular disease (ASCVD) are closely related to the severity of coronary artery calcification (CAC)^3^. The main process of vascular calcification involves the osteogenic differentiation of arterial smooth muscle cells (SMCs) into osteoblast-like cells^4^. In response to various factors, such as transforming growth factor-β1 (TGF-β1)^5^, tumor necrosis factor-alpha (TNF-α)^6^, β-glycerophosphate^7^ and reactive oxygen species^7^, VSMCs express osteogenic factors, including runt-related transcription factor 2 (Runx2) and osteopontin^8^. However, there are currently no therapeutic options that are clinically available to prevent vascular calcification^9^.

Macrophages are the major immune cell population that is involved in cardiac remodeling after myocardial infarction, and they participate in this process by producing pro- and anti-inflammatory cytokines, such as TNF-α, IL-1β and TGF-β1^10^. They are also present in atherosclerotic lesions and play critical roles in vascular calcification by secreting chemokines/cytokines^11^. Macrophages coexist with calcium deposits and accelerate calcification^12^, inducing the osteogenic differentiation of vascular smooth muscle cells^12^. It was previously reported that macrophages release calcifying matrix vesicles (MVs), which are a kind of EV that is enriched in S100A9 and annexin V, and MVs contribute to vascular calcification in chronic renal disease^13^. However, the role of macrophage-derived EVs in the pathogenesis of vascular calcification is largely unknown^14^. Furthermore, to the best of our knowledge, no study has reported the effects of miRNAs in EVs derived from macrophages on vascular calcification under high glucose conditions.

Extracellular vesicles (EVs) are small vesicles that are released from most cell types and can be isolated from almost all biological fluids including culture media. The main subtypes of EVs include exosomes and microvesicles. Exosome originate from intracellular vesicles, typically formed through the endosome system. On the other hand, microvesicles are often released directly from the cell membrane. They incorporate various molecules, such as proteins, lipids, and nucleic acids [mainly microRNAs (miRNAs)]^15–17^. These contents vary according to the state of the parental cells, thus reflecting the cellular context^15^. Several studies have reported that EVs participate in multiple diseases, including cancer^18^, diabetes mellitus^19^, and cardiovascular diseases^20–22^, by transferring miRNAs to recipient cells. miRNAs are noncoding and single-stranded RNAs that are composed of 19–25 nucleotides. They posttranscriptionally modulate gene expression by binding to the 3’-UTRs of target mRNAs and regulating the stability and/or translation of these mRNAs^23^. Accumulating evidence has suggested that miRNAs could be a new therapeutic target in various diseases (e.g., cancers^24^), but the role of EVs in vascular calcification is largely unknown^25^.

In the present study, we performed miRNA screening on macrophage-derived EVs and sought to examine whether an miRNA in EVs that was identified regulates the osteogenic differentiation of VSMCs under high glucose conditions.

## Results

### Characterization of EVs secreted by macrophages under normal or high glucose conditions

**Figure 1:**
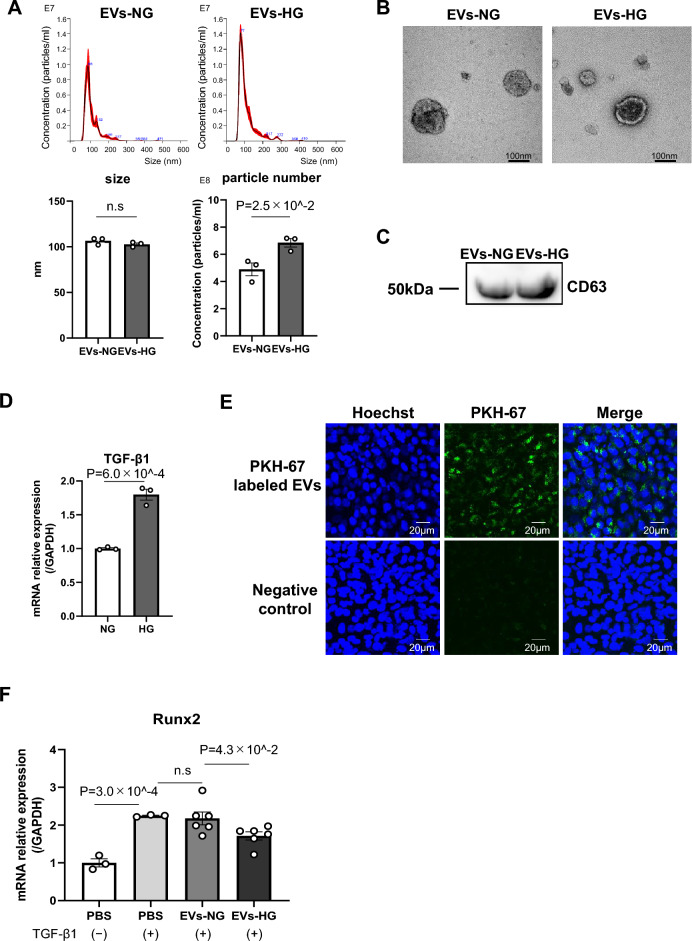
EVs-HG repressed the TGF-β1-induced transdifferentiation of VSMCs. (**A–C**) Characterization of EVs secreted by macrophages under normal glucose and high glucose conditions. Representative graphs of extracellular vesicles from macrophages using Nano tracking analysis. Particle size and concentration of EVs from macrophages exposed to 5 mM (NG) or 25 mM (HG) glucose conditions (**A**) (N = 3). Electron microscopy of EVs-NG or EVs-HG, showing small vesicles around 100 nm in diameter (B). Western blotting analysis was performed to show the expression of CD63, a representative EVs’ surface antigen, in EVs-NG and EVs-HG (**C**). Original blots/gels are presented in Supplementary Figure S1. (**D**) The expression level of TGF-β1 in primary macrophages cultured under normal glucose or high glucose conditions was evaluated by RT‒PCR (N = 3). (**E**) Macrophage-derived EVs were internalized by VSMCs. EVs (concentration of 2.0 × 10^9 particles/mL) were labeled with PKH-67 (green fluorescent marker) and incubated with MOVAS cells for 24 h. Hoechst 33342 (blue fluorescent marker) was used to stain the nuclei, and cells were photographed using fluorescence confocal microscopy. (**F**) MOVAS cells were treated with EVs-NG or EVs-HG (concentration of 2.0 × 10^9^ particles/mL) for 24 h, followed by 2 ng/ml TGF-β1 treatment for 6 h. EVs-HG repressed the TGF-β1-mediated induction of Runx2 expression at the transcript level in MOVAS cells. GAPDH was used as an endogenous control (N = 6). The P value was calculated by unpaired Student’s t test, and the data are presented as the mean ± SEM.

We first examined whether high glucose conditions could affect EV release from macrophages. Vesicles were isolated from the supernatants of peritoneal macrophages that were exposed to normal glucose and high glucose conditions. Nano tracking analysis showed that high glucose increased the number of vesicles that were released from macrophages compared to normal glucose. On the other hand, both groups showed similar vesicle sizes with diameters in the range of 100–120 nm (Fig. 1A). The morphology of the vesicles was characterized by transmission electron microscopy (TEM). We identified cup-shaped particles with diameters of 100–120 nm, and these features are consistent with previous reports on EVs^15^ (Fig. 1B). Analysis of equal volumes of samples by western blotting revealed that both EVs-NG and EVs-HG expressed the CD63 protein, which is an surface antigen of EVs (Fig. 1C). These results indicated that macrophages released small vesicles with characteristics that are compatible with EVs, and high glucose conditions did not alter the morphology of the EVs but rather increased the number of EVs secreted.

### High glucose conditions increased TGF-β1 expression in macrophages

Since TGF-β1 is the major cytokine that is involved in diabetic complications, including vascular calcification^5,26^, and macrophages play essential roles in vascular calcification in part by secreting osteogenic cytokines, we assumed that high glucose conditions could influence TGF-β1 expression in macrophages. We then exposed primary macrophages to normal glucose or high glucose conditions for 48 h and assessed the gene expression of TGF-β1 by real-time PCR. As expected, high glucose increased TGF-β1 gene expression compared with normal glucose (Fig. 1D). This result suggested that high glucose conditions could promote the osteogenic differentiation of VSMCs by increasing TGF-β1 production by macrophages.

### EVs-HG inhibited the TGF-β1-induced osteogenic differentiation of VSMCs

EVs are mediators of signal transduction in disease progression^18–20^. Therefore, we examined the effects of EVs secreted by macrophages under high glucose conditions on the osteogenic differentiation of VSMCs. First, we examined whether MOVAS cells could take up macrophage-derived EVs. After 24 h of incubation, PKH67-labeled EVs (concentration of 2.0 × 10^9 particles/mL) were taken up by MOVAS cells (Fig. 1E), indicating that VSMCs took up macrophage-derived EVs. We next sought to investigate the effect of macrophage-derived EVs on the TGF-β1-induced osteogenic differentiation of VSMCs. To this end, we incubated MOVAS cells with EVs-NG or EVs-HG for 24 h and treated the cells with TGF-β1 (2 ng/mL). Consistent with previous reports, TGF-β1 increased the gene expression of Runx2^27^, which is an essential and sufficient regulator of calcification of VSMCs^28^, and EVs-HG significantly inhibited the gene expression of Runx2 compared to EVs-NG (Fig. 1G). These data suggested that EVs that were secreted by macrophages under high glucose conditions inhibited the TGF-β1-induced osteogenic differentiation of VSMCs.

### Microarray analysis of miRNAs in EVs identified miR-17-5p as a potential miRNA that is involved in vascular calcification by modulating the TGF-β signaling pathway

EVs contribute to cell signaling by transporting noncoding RNAs, including miRNAs. To investigate the molecular mechanisms by which EVs-HG affect the TGF-β1-induced osteogenic differentiation of VSMCs, we explored the miRNA expression profiles in a comprehensive manner. To this end, a mannitol group (EVs-mannitol) was also added as an osmotic control for high glucose conditions because hyperosmotic stress may influence the miRNA expression levels.

**Figure 2:**
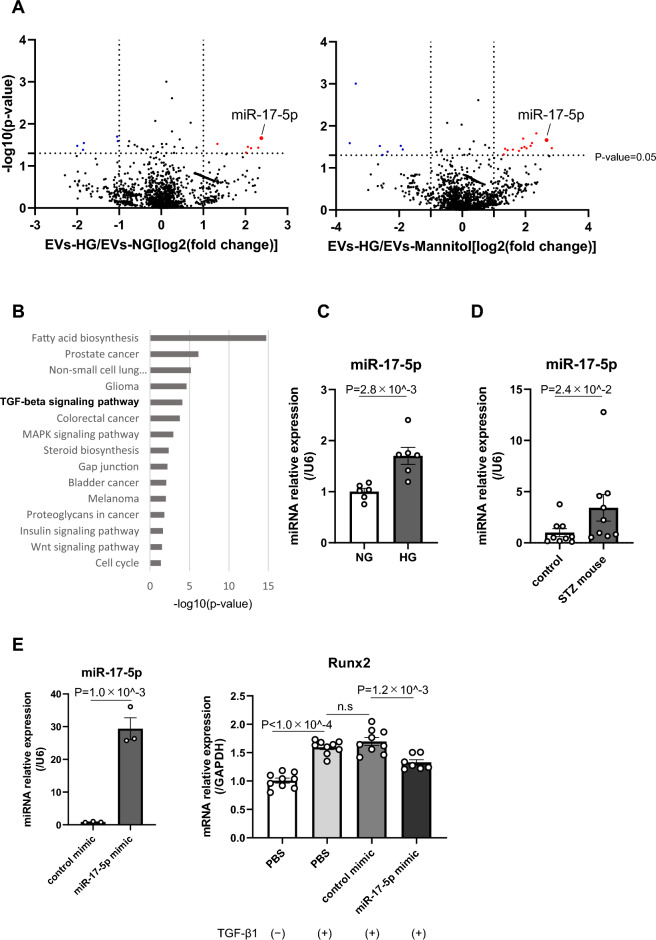
miR-17-5p repressed the TGF-β1-induced transdifferentiation of VSMCs. (**A**) Volcano plot of miRNA expression in EVs. The plot shows the intensity of miRNA expression between EVs-HG and EVs-NG (left panel) and EVs-HG and EVs-mannitol (right panel). The horizontal axis represents the log2 (EVs-HG/EVs-NG fold change) or log2 (EVs-HG/EVs-mannitol fold change). The vertical axis represents –log10 (*p* value). The black dashed line on the vertical axis refers to the threshold value corresponding to a corrected p value of *p* = 0.05. Red indicates miRNAs that were significantly increased by more than twofold in EVs-HG, and blue indicates miRNAs that were significantly decreased by more than twofold in EVs-HG. (**B**) Pathway analysis using DIANA TOOLS-mirPath v.3 software (TarBase v7.0). KEGG pathways determined in mmu-miR-17-5p are shown. (**C**) Validation of the expression of miR-17-5p identified in the microarray using RT‒PCR. Macrophages were cultured under normal glucose or high glucose conditions for 48 h, and the expression levels of miR-17-5p were quantified using RT‒PCR (N = 6). (**D**) miR-17-5p expression level analysis in a diabetes mouse model. The serum expression levels of miR-17-5p were measured by RT‒PCR in a mouse model of STZ-induced diabetes, showing significantly higher expression than in nondiabetic mice (N = 9). (**E**) Relative expression of miR-17-5p in MOVAS cells after transfection with miR-17-5p mimic (10 nM) or mimic control (N = 3). miR-17-5p mimic or mimic control was transfected, and 2 ng/ml TGF-β1 was added. Runx2 expression levels were measured by RT‒PCR (N = 9). GAPDH or U6 was used as the endogenous control. The *p* value was calculated by one-way ANOVA followed by a post hoc test (**A**), Mann‒Whitney U test (**D**), and unpaired Student’s t test. The data are presented as the mean ± SEM.

We performed a microarray of miRNAs in EVs among the EVs-NG, EVs-HG, and EVs-mannitol (an osmotic control for high glucose conditions) groups. We found that 6 miRNAs (mmu-miR-17-5p, 7660-5p, 350-3p, 376b-3p, 6956-3p, and 3083-5p) among 1963 miRNAs included in the Oligo Chip were significantly increased and 5 miRNAs (mmu-miR-1981-5p, 6967-5p, 7072-3p, 34c-3p, and 6967-3p) were significantly decreased in EVs-HG, and their expression levels differed from those in EVs-NG by at least twofold. mmu-miR-17-5p was the most differentially expressed miRNA among these molecules (fold change = 5.19) (Fig. 2A, left panel). To exclude the possibility that hyperosmotic stress under high glucose conditions could influence miR-17-5p expression, we also compared differentially expressed miRNAs between EVs-HG and EVs-mannitol. miR-17-5p was also differentially expressed in EVs-HG compared to EVs-mannitol [fold change = 6.37, Fig. 2A (Right panel)], suggesting that the hyperosmotic state did not affect miR-17-5p expression. We further performed pathway analysis of miR-17-5p using DIANA-miR-Path and showed that miR-17-5p target gene pathways included “fatty acid biosynthesis” (*p* value = 1.83E-15) and “TGF-β signaling pathway” (*p* value = 7.96E-05) (Fig. 2B). As mentioned above, TGF-β1 is the major cytokine that is involved in diabetic complications, including vascular calcification^5,26^, we hypothesized that miR-17-5p would be one of the key miRNAs that contributes to vascular calcification by modulating the TGF-β signaling pathway.

Next, we validated miR-17-5p expression in donor macrophages to support the microarray results. Under high glucose conditions, peritoneal macrophages exhibited increased miR-17-5p expression (Fig. 2C), suggesting that EVs-HG contained higher levels of miR-17-5p than EVs-NG. Furthermore, we examined the serum levels of miR-17-5p in mice with STZ-induced diabetes. Mice with consistently elevated blood glucose levels (> 300 mg/dL) were considered diabetic (Supplementary Figure S2). The serum levels of miR-17-5p significantly increased in mice with STZ-induced diabetes compared with control mice (Fig. 2D), suggesting the potential link between serum levels of miR-17-5p and high glucose conditions. Considering that miRNA in the serum is generally encapsulated within EVs, this data suggested that miR-17-5p would be enriched in EVs-HG. Therefore, we focused on the functions of miR-17-5p in the osteogenic differentiation of VSMCs.

### miR-17-5p inhibited the TGF-β1-induced osteogenic differentiation of VSMCs

We investigated the effect of miR-17-5p on TGF-β1-induced VSMC osteogenic differentiation. We transfected the miR-17-5p mimic into MOVAS cells using lipid nanoparticles (Fig. 2E) and assessed the effect of miR-17-5p on the expression of osteogenic genes. Consistent with the data that macrophage-derived EVs-HG inhibited the TGF-β1-induced osteogenic differentiation of VSMCs, overexpression of miR-17-5p inhibited the mRNA levels of Runx2 in MOVAS cells compared with the control groups (Fig. 2E, right panels).

### TGF-β RII is a potential target of miR-17-5p and EVs-HG suppressed TGF-β RII expression in VSMCs partly through EV-mediated transfer of miR-17-5p

**Figure 3:**
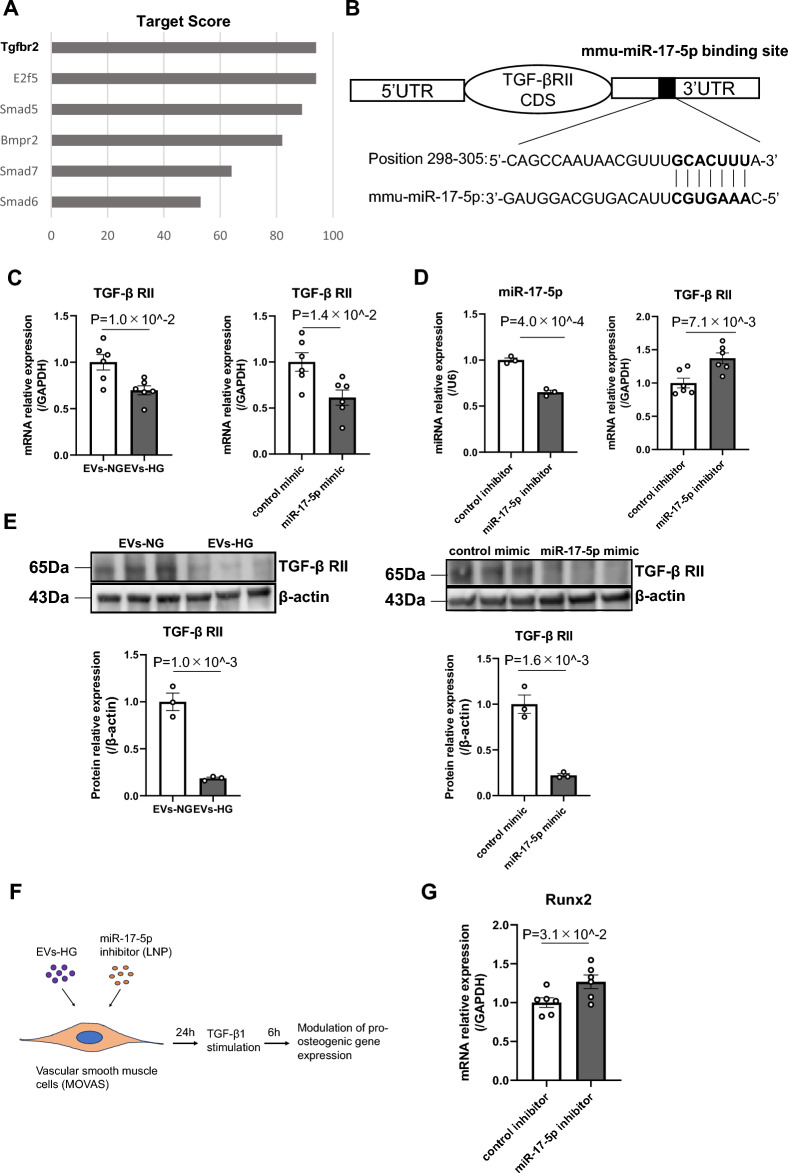
EVs-HG suppressed the expression of TGF-β RII partly through transferring miR-17-5p. (**A**) TGF beta-related target genes for mmu-miR-17-5p were identified by the miRBase online database (https://www.mirbase.org). A higher score represents more statistical confidence in the prediction result. (**B**) Illustration of the potential base-pairing sites of miR-17-5p located in the 3’-UTR of TGF-β RII mRNA. (**C**) Analysis of TGF-β RII expression levels in MOVAS cells transfected with EVs-NG or EVs-HG and control mimic or miR-17-5p mimic. EVs-HG and miR-17-5p mimic significantly decreased TGF-β RII expression at transcript level respectively compared to EVs-NG or control mimic (N = 6). (**D**) The level of miR-17-5p was silenced in MOVAS cells by transfection of miR-17-5p inhibitor (N = 3). Inhibiting miR-17-5p in MOVAS cells significantly increased the TGF-β RII transcript levels compared to control inhibitor (N = 6). (**E**) EVs-HG and elevated expression of miR-17-5p reduced TGF-β RII expression at protein level respectively compared to EVs-NG or control mimic (N = 3). Original blots/gels are presented in Supplementary Figure S3−S4. (**F**) Schematic representation of the treatment of Fig. 3G is shown. (**G**) The gene expression of Runx2 was evaluated in the presence of 2 ng/ml TGF-β1. Inhibiting miR-17-5p in MOVAS cells cotransfected with EVs-HG significantly increased the Runx2 transcript levels (N = 6)**.** GAPDH, β-actin, or U6 was used as the endogenous control. The *p* value was calculated by unpaired Student’s t test, and the data are presented as the mean ± SEM.

MicroRNAs regulate the stability and/or translation of mRNAs by binding to the 3’-UTRs of target mRNAs. Therefore, we predicted the potential biological target of miR-17-5p using miRBase to investigate the mechanism by which the miR-17-5p mimic inhibited TGF-β1-induced osteogenic differentiation. Among 966 candidate genes, six potential target genes related to the TGF-β signaling pathway were identified based on target scores by miRbase^29^ (Fig. 3A). miRBase predicted the sequence-specific base pairing sites in the 3’ untranslated region of TGF-β RII (Fig. 3B). Since TGF-β1 initiates its signal by binding to two receptors (type I and type II receptor) and triggering downstream Smad 2/3 and Smad 4 activation, we focused on TGF-β receptor II (TGF-β RII) as a potential target of miR-17-5p. EVs-HG contained higher levels of miR-17-5p than EVs-NG (Fig. 2C), therefore, we further validated our prediction to determine whether EVs-HG and miR-17-5p could inhibit TGF-β RII. Incubation of MOVAS cells with EVs-HG, and miR-17-5p overexpression by transfection of the miR-17-5p mimic using lipid nanoparticles significantly decreased TGF-β RII expression at mRNA level (Fig. 3C). On the other hand, inhibition of miR-17-5p using miR-17-5p antagomir-containing lipid nanoparticles increased TGF-β RII expression at mRNA level (Fig. 3D). We also showed the inhibitory effect of TGF-β RII expression by EVs-HG and miR-17-5p at protein level (Fig. 3E), suggesting that TGF-β RII might be a potential target of miR-17-5p in VSMCs and EVs-HG could inhibit TGF-β RII expression partly through transferring of miR-17-5p. Finally, we verified the expression level of Runx2 by miR-17-5p inhibitor and demonstrated that inhibition of miR-17-5p in EVs-HG using miR-17-5p antagomir-containing lipid nanoparticles increased TGF-β1-induced Runx2 gene expression in MOVAS cells (Fig. 3G).

### miR-17-5p inhibited calcium deposition in VSMCs

**Figure 4:**
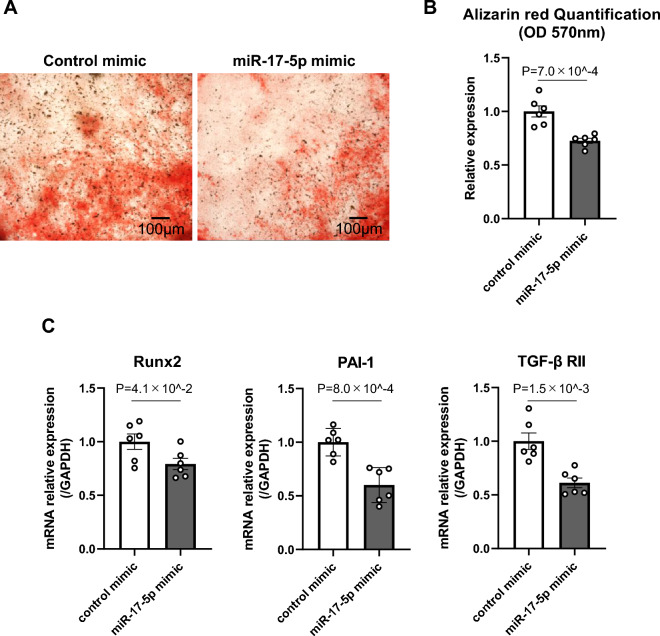
miR-17-5p inhibited VSMC calcification. (**A**, **B**) Transfection of miR-17-5p inhibits calcium deposition. VSMCs were incubated with calcification medium in the presence of miR-17-5p mimic or control mimic. Calcium deposition was determined by Alizarin Red S staining at 7 days, demonstrating the effect of miR-17-5p on VSMC calcification (**A**). Quantification was also performed by measuring the absorbance at a wavelength of 570 nm by a spectrophotometer (**B**) (N = 6). (**C**) Runx2, PAI-1, and TGF-β RII expression levels were measured by RT‒PCR. GAPDH was used as the endogenous control (N = 6). The P value was calculated by unpaired Student’s t test, and the data are presented as the mean ± SEM.

Finally, we investigated whether the miR-17-5p mimic could inhibit the calcification of VSMCs to test miR-17-5p’s potential as a therapeutic agent for osteogenic differentiation of VSMCs. Since high-phosphate medium induces the osteogenic differentiation of VSMCs increasing Runx2 expression (Supplementary Figure S5) and TGF-β signaling is significantly activated in VSMCs cultured with high-phosphate media^27,30^, MOVAS cells were cultured in high-glucose and high-phosphate media for 7 days. After transfecting the miR-17-5p mimic into the MOVAS cells, we showed that overexpression of miR-17-5p suppressed calcium deposition as assessed by alizarin red staining (Fig. 4A,B). We also showed that the miR-17-5p mimic inhibited TGF-β RII, Runx2, and PAI-1 expression compared to the control mimic in high-glucose and high-phosphate media (Fig. 4C).

## Discussion

In this study, we elucidated the roles of macrophage-derived EVs and miRNA in vascular calcification under high glucose conditions. The key findings include the following: (1) High glucose increased the secretion of macrophage-derived EVs that inhibit the TGF-β1-induced osteogenic differentiation of VSMCs; (2) miRNA array analysis of EVs identified miR-17-5p as a potential miRNA that is involved in vascular calcification by modulating the TGF-β signaling pathway; and (3) miR-17-5p lipid nanoparticles mimicked EVs-HG by inhibiting TGF-β RII and the osteogenic differentiation of VSMCs.

Recently, the pathogenic or therapeutic roles of EVs and miRNAs have begun to attract attention, and researchers have focused on EV-based therapies and their clinical applications. In vascular calcification, previous studies have shown that several miRNAs could play essential roles in the regulation of VSMC osteogenic differentiation. miR-223-3p may inhibit the osteogenic switch of vascular smooth muscle cells by regulating IL-6/STAT3 signaling^31^, and miR-103a may regulate the calcification of vascular smooth muscle cells by targeting the expression of Runx2 under high phosphorus conditions^32^. In the present study, we showed that miR-17-5p inhibits the TGF-β1-induced osteogenic differentiation of VSMCs by negatively modulating TGF-β RII expression. This was consistent with previous studies that were reported by other investigators that showed miR-17-5p serves as an important negative regulator of the TGF-β signaling pathway by inhibiting TGF-β RII ^33–36^.

In the clinical setting, plasma levels of miR-17-5p were increased in women with gestational diabetes mellitus (GDM)^37^, and another study showed that plasma miR-17-5p expression was increased in type 2 diabetes mellitus (T2DM) patients with nonalcoholic fatty liver disease (NAFLD)^38^. miR-17-5p was also reported to be a novel biomarker for the diagnosis of acute myocardial infarction^39^ and for the severity of coronary atherosclerosis in coronary artery disease^40^. These studies suggested that miR-17-5p may be associated with diabetes mellitus and atherosclerotic lesion development; however, the pathogenic roles of miR-17-5p are largely unknown. Our study showed for the first time the protective role of miR-17-5p in the context of diabetic vascular calcification.

We showed that the level of TGF-β1, which plays a crucial role in vascular calcification^5^, was increased in macrophages that were exposed to high glucose conditions. These data were consistent with a previous report showing that high glucose conditions increased the secretion of the TGF-β1 protein from murine macrophages in a concentration-dependent manner^41^. Additionally, our study demonstrated for the first time that high glucose conditions elevated the expression level of miR-17-5p in both macrophages and macrophage-derived EVs. As shown in several reports, miR-17-5p directly targets TGF-β RII and regulates its expression, inhibiting the TGF-β signaling pathway^33–36^. Taken together, these results show that while macrophages promote VSMC calcification by secreting TGF-β1 under high glucose conditions, EVs enriched in miR-17-5p may attenuate the TGF-β signaling that is activated by high glucose via the inhibition of TGF-β RII expression. Indeed, some previous studies have reported that specific miRNA expression was increased under pathological conditions and that these miRNAs simultaneously play roles as essential mediators of negative feedback loops to control pathogenesis^42^. Considering that a prolonged and exaggerated response to TGF-β1 is detrimental, it is reasonable that EVs whose secretion is induced by high glucose could negatively regulate TGF-β signaling by delivering inhibitory miRNAs to VSMCs.

miRNAs control gene expression and can be a promising therapy for diverse diseases, but at the same time, miRNAs are easily degraded by RNase both in vitro and in vivo. In addition, miRNAs alone cannot pass through cell membranes^43^. Therefore, we used lipid-based nanoparticles (a kind of drug delivery system that is also considered to be safe for use in animals)^44^ to efficiently deliver miRNAs into cells. In the present study, VSMCs transfected with miR-17-5p mimic exhibited diminished calcium deposition. TGF-β RII and TGF-β signaling have been demonstrated to be increased in CKD arteries and VSMCs cultured with high-phosphate media, leading to VSMC calcification^30,45^. Additionally, PAI-1 expression is increased by TGF-β1, which exerts pro-calcific effects^46^, and inhibition of TGF-β/PAI-1 signaling with an anti-TGF-β antibody could suppress phosphate-induced vascular calcification^46^. These findings suggest that the miR-17-5p mimic ameliorated high-phosphate-induced VSMC calcification by interfering with TGF-β signaling. Nanoparticle-mediated delivery of miR-17-5p to VSMCs could be applicable for the prevention and treatment of vascular calcification in the future.

Very recently, Koide et al. showed that miR-17-5p mitigated vascular calcification by inhibiting VEGF-A in a chronic kidney disease (CKD) model^47^. miRNAs have multiple target genes. Since the profiles of important genes expressed vary depending on the disease state, it is necessary to validate the effectiveness and off-target effects of miR-17-5p in each disease state. Nonetheless, exogenous treatment with miRNAs is positioned to enhance endogenous protective mechanisms as a therapeutic strategy.

There are some limitations in the present study. We showed calcium deposition by Alizarin red staining at seven days. Further investigations will be needed to evaluate over a longer period or using alternative methods such as Von Kossa staining. In addition to this, arterial calcification in the diabetic mice has not been verified. Future in vivo studies will address whether miR-17-5p could also inhibit VSMCs calcification, validating the mechanisms underlying the association between macrophage-derived EVs and vascular calcification under high glucose conditions. Furthermore, there is a possibility that miRNAs other than miR-17-5p, or other molecules may contribute to these results. For example, macrophages also express E-cadherin^48^ and/or N-cadherin^49^, which are known to regulate osteogenic differentiation^50^. We should consider that EVs-associated cadherin may have influenced these results, and directly inhibiting molecules like cadherin could be effective in treating calcification. However, in our study, we identified miR-17-5p through unbiased screening of miRNAs and our in vitro experiments using a miR-17-5p mimic and inhibitor validate that miR-17-5p is at least partially involved in the TGF-β signaling for osteogenic differentiation of VSMCs, and can be a therapeutic agent for regulating calcification.

In conclusion, the present study demonstrated several lines of novel evidence that EVs that are derived from macrophages under high glucose conditions effectively suppress VSMC calcification partly through the EV-mediated transfer of miR-17-5p. miR-17-5p could be a potential therapeutic agent for vascular calcification in the future.

## Methods

### Animals

Male C57BL/6 mice, weighing 20–26 g and aged 6–12 weeks, were housed in pathogen-free cages under conditions of a constant room temperature (22 ± 2 °C), constant humidity (40–60%), and a 12-h light/dark cycle. In all the procedures, animals were anaesthetized by 2% isoflurane inhalation, and after all the experiments, the mice euthanized by cervical dislocation following 5% isoflurane inhalation. All the animal experimental procedures in this study were approved by the Kyushu University ethics committee in advance, and were carried out following the ARRIVE guidelines. The experiments were also complied with the Administrative Instructions of Kyushu University Animal Experiment Regulations, the Fundamental Guidelines for Animal Experiments and Related Activities (the Japanese Ministry of Education, Culture, Sports, Science and Technology), and the Guidelines for Proper Conduct of Animal Experiments (the Science Council of Japan) as instructed by the university.

### Cell culture

Mouse VSMC cell line MOVAS (ATCC-CRL-2797, USA) was cultured in high-glucose Dulbecco’s modified Eagle’s medium (DMEM) supplemented with 10% foetal bovine serum, 1% penicillin, and 1% streptomycin. The cells were cultured at 37 °C in a humidified atmosphere with 5% CO_2_. The growth medium was refreshed every 3 days, and cells between passages 3 and 9 were used for subsequent experiments.

Peritoneal macrophages were isolated from mice that were intraperitoneally injected with thioglycolate as previously described^51^. Isolated peritoneal macrophages were treated with RPMI-1640 medium supplemented with 5 mM glucose for 24 h and then divided into 2 groups: the normal glucose (NG) group (macrophages were treated with 5 mM glucose) and high glucose (HG) group (macrophages were treated with 25 mM glucose). In miRNA array analysis, we also included a mannitol group (5 mM glucose + 20 mM mannitol) as an osmotic control for high glucose treatment. EV-depleted FBS was used to supplement the cell culture medium, and after being cultured for another 48 h, EVs were isolated from the culture supernatants as described below.

### Isolation of EVs

Purification of EVs from medium supernatant of Macrophage (10mL) with the Tim4-affinity method was performed using the MagCapture™ Exosome Isolation Kit PS (FUJIFILM Wako Chemicals U.S.A. Corp) following the manufacturer’s protocol. In brief, supernatant samples were centrifuged at 1200 g for 20 min at 4 °C to remove cell debris and then centrifuged at 10,000 g for 30 min at 4 °C to remove the large EVs such as apoptotic bodies. Streptavidin Magnetic Beads, bound with biotinylated Tim 4, were added to the supernatant samples supplemented with 2 mM CaCl_2_, and the mixture was rotated for 3 h at 4 °C. The beads were washed three times with washing buffer (20 mM Tris–HCl, pH 7.4, 150 mM NaCl, 0.0005% Tween 20, 2 mM CaCl_2_), and the EVs bound with beads were eluted with elution buffer (20 mM Tris–HCl, pH 7.4, 150 mM NaCl, 2 mM EDTA). EVs were divided into two groups, namely, the EVs-NG group (EVs extracted from macrophages treated with 5 mM glucose) and EVs-HG group (EVs extracted from macrophages treated with 25 mM glucose), and stored at − 70 °C until use.

### Identification of EVs

To identify EVs, 1) nanoparticle tracking analysis was performed to evaluate the size and concentration of the macrophage-derived EVs using a NanoSight NS 300 device (Malvern Panalytical Ltd, Worcestershire UK), 2) the morphology of the purified EVs was determined by electron microscopy analysis supported by Hanaichi Ultrastructure Research in Japan, and 3) the expression of the EVs’ surface marker antigen CD63 (EXOAB-CD63A-1, SYSTEM BIOSCIENCES, USA) was analyzed by western blotting analysis. Details of the NTA measurement were shown as below: camera level was increased until particles were distinctly visible (level 12). For each measurement, five 1-min videos were captured under the following conditions: temperature: 25 °C; Syringe speed: 200μL/sec. After capture, the videos have been analysed by the NanoSight Software with a detection threshold of 5.

### Analysis of Exo-miRNA expression

To compare miRNA expression among EVs-NG, EVs-HG and EVs-mannitol, comprehensive miRNA-expression analysis including labeling, hybridization, scanning, and data processing were all performed by TORAY Industry (Kanagawa, Japan) using the 3D-Gene Mouse miRNA Oligo Chip ver.21. This chip contains 1900 antisense probe spots, and miRNAs with positive signals that were higher than background were selected and statistically analyzed among the groups. The global mean normalization method was used to standardize the miRNA array data (log conversion of data and median alignment), and the expression levels in three samples from each EV group were assessed to avoid differences among individual samples.

### Analysis of EV uptake by VSMCs

EVs were labeled with a PKH-67 fluorescent cell linker kit (PKH67GL, Sigma‒Aldrich, USA), and unincorporated dye contamination was removed using Exosome Spin Columns (MW3000, Invitrogen, USA) according to the manufacturer’s instructions. The labeled EVs were incubated with VSMCs at 37 °C for 24 h. Then, Hoechst 33342 Staining Solution (NucBlue^TM^Live Cell Stain ReadyProbesTM reagent, Thermo Fisher Scientific, USA) was added and incubated for 30 min, and the staining signals were analyzed with a fluorescence microscope (FV1200, IX83, Olympus Life Science, Japan).

### miRNA transfection

To transfect miRNAs, mirVana miRNA mimic or inhibitor reagents [miRNA Mimic negative control (#4464058), miR-17-5p mimic (#4464066), miRNA Inhibitor negative control (#4464076), and miR-17-5p inhibitor (#4464084)] were purchased from Thermo Fisher Scientific (USA) and encapsulated with lipid-based nanoparticles as previously reported^44^. Briefly, miRNAs were prepared in HEPES buffer (pH 5.0–5.4) and encapsulated into ab envelope formed with ssPalm (Product# COATSOME®SS-OP, NOF CORPORATION, Tokyo, Japan) and helper lipids with vigorous mixing. The mixed solution was further diluted with HEPES and concentrated by centrifugation at 1000 × g for 20 min using an Amicon Ultra filter (Millipore Corp. Billerica, MA). Finally, the particle solution that remained in the upper column was diluted with PBS, and then, 10 nM miRNA was transfected into MOVAS cells.

### STZ-induced diabetes mouse model

A low-dose streptozotocin induction protocol was used to establish a diabetes mouse model as previously reported^52^. In brief, male C57BL/6J mice were intraperitoneally injected with 50 mg/kg streptozotocin (S0130, Sigma‒Aldrich) dissolved in 0.1 M sodium citrate buffer at pH 4.5. Each mouse was administered streptozotocin for 5 consecutive days. Control (nondiabetic) mice received injections of buffer alone. The development of diabetes was monitored by measuring blood glucose levels (FreeStyle, NIPRO, Japan), and only mice with consistently elevated blood glucose levels (> 300 mg/dL) were considered to have diabetes and used in further studies. At 4 weeks after STZ administration, 1 ml of blood volume per mouse was collected by directly puncturing the inferior vena cava. Then, 500 μL of plasma sample was isolated from whole blood by centrifugation at 3000 g for 15 min at 4 °C and stored at − 80 °C.

### Real-time reverse-transcription polymerase chain reaction

RNeasy Kits (QIAGEN) and the mirVana™ miRNA Isolation Kit (Thermo Fisher Scientific) were used to extract mRNA and miRNA from cultured cells, respectively. The miRNeasy Serum/Plasma Kit (QIAGEN) was used to purify miRNA from plasma samples of animal models. Then, the extracted RNA was reverse transcribed into cDNA using the PrimeScript™ RT Reagent Kit (TAKARA BIO INC) for mRNA and the TaqMan™ miRNA Reverse Transcription Kit (Thermo Fisher Scientific) for miRNA. Then, SYBR Green RT‒PCR Reagents (Applied Biosystems by Thermo Fisher Scientific) were used to perform quantitative PCR. The reaction was conducted with a StepOne™ Real-Time PCR System (Thermo Fisher Scientific). The mRNA expression results were normalized to GAPDH expression. U6 expression served as an internal control for miRNA expression. We used the 2^-ΔΔCt^ method to calculate the fold changes in expression. The nucleotide sequences of the primers used in this study are available in the Supplementary Table.

### Western blot analysis

Protein extraction was performed using RIPA buffer (Nacalai Tesque, Japan). Then, the protein concentration was measured with a Pierce^TM^BCA Protein Assay Kit (Thermo Fisher Scientific). Twenty-five micrograms of lysates were resolved by SDS‒PAGE and transferred to PVDF membranes using the Trans-Blot Turbo Transfer System (Bio-Rad Laboratories) (Technical limitation: in the detection of CD63 band, we added an equivalent volume not an equal amount of what was collected using the kit because the amount of protein in the EVs was technically difficult to quantify due to its small amount). The membranes were blocked with nonfat dry milk solution for 1 h and then incubated overnight with mouse monoclonal primary antibodies against TGF-β RII (1:1000; sc-17799, Santa Cruz Biotechnology, USA) in blocking buffer at 4 °C. After three washes with TBS-T the, membranes were incubated with sheep anti-mouse peroxidase-conjugated secondary antibody (1:5000; GE Healthcare). β-actin HRP (1:30,000; a3854, Sigma Aldrich) was used as an internal control. The blots were visualized using enhanced chemiluminescence reagent (RPN2235, Cytiva, USA) with an imaging system (ImageQuantTM LAS4010, Cytiva, USA). We cut the blots prior to hybridization with antibodies so that the images of full-length blots were not provided.

### Induction of osteogenic differentiation in VSMCs

Osteogenic differentiation of VSMCs was induced with high-phosphate medium as previously reported^53^. Briefly, MOVAS cells were routinely subcultured in normal growth medium as described above. After reaching confluence, the cells were switched to calcification medium consisting of normal growth medium supplemented with 5 mM Pi [a mixture of NaH_2_PO_4_ and Na_2_HPO_4_ (pH 7.4)], 50 μg/mL ascorbic acid and 2.7 mM CaCl_2_. The medium was replaced with fresh medium every 3 to 4 days for 7 days.

### Alizarin Red S staining

Cells were seeded into 96-well plates and cultured in osteogenic medium for 7 days. The culture plate was fixed with 10% formaldehyde for 60 min at room temperature and stained with alizarin Red S solution (2% aqueous solution, pH = 4.1–4.3) for 30 min. The cells were then fully washed with distilled water and observed under a microscope. Positive staining is shown as red colour areas. For quantification, the absorbance was measured at a wavelength of 570 nm by a spectrophotometer (xMark™ Microplate Spectrophotometer, Bio-Rad Laboratories, USA).

### Statistical analysis

All the data are presented as the mean ± standard error of the mean (SEM) and were analyzed using Prism software (GraphPad Prism V.8.). Paired comparisons between groups were carried out by Student’s t test for normally distributed data from at least three replicates. The Shapiro‒Wilk test was used to test the normality of variables, and when the data were not normally distributed, we used the nonparametric Mann‒Whitney U test. In miRNA array analysis, comparisons among three groups were performed using one-way analysis of variance (ANOVA) followed by a post hoc test. Representative experimental data are shown in the figures, and statistical significance was defined as *p* values < 0.05.

### Supplementary Information

Supplementary Figures.

Supplementary Table 1.

## Data Availability

The data that support the findings of this study are available within the article and its supplementary information files.
