# Author Correction: EVs-miR-17-5p attenuates the osteogenic differentiation of vascular smooth muscle cells potentially via inhibition of TGF-β signaling under high glucose conditions

**DOI:** 10.1038/s41598-024-70722-x

**Published:** 2024-08-23

**Authors:** Isashi Baba, Tetsuya Matoba, Shunsuke Katsuki, Jun-ichiro Koga, Takuro Kawahara, Mitsukuni Kimura, Hidetaka Akita, Hiroyuki Tsutsui

**Affiliations:** 1 Department of Cardiovascular Medicine, Graduate School of Medical Sciences, Kyushu University, 3-1-1, Maidashi, Higashi-ku, Fukuoka, 812-8582 Japan; 2 Second Department of Internal Medicine, School of Medicine, University of Occupational and Environmental Health, Kitakyushu, Japan; 3 Laboratory of Drug Design and Drug Disposition, Graduate School of Pharmaceutical Sciences, Tohoku University, Sendai, Japan

Correction to: *Scientific Reports* 10.1038/s41598-024-67006-9, published online 15 July 2024

The original version of this Article contained a repeated error in the text and Figure 2 legend, where

“Exo-mannitol”

now reads:

“EVs-mannitol”

The original Article has been corrected.

