# Supplementary material for: EVs-miR-17-5p attenuates the osteogenic differentiation of vascular smooth muscle cells potentially via inhibition of TGF-β signaling under high glucose conditions
Source: Sci Rep. 2024 Jul 15;14:16323. doi: 10.1038/s41598-024-67006-9 (PMC11251274; doi:10.1038/s41598-024-67006-9)
Supplement: Supplementary file 1 — Supplementary Figures. [file 41598_2024_67006_MOESM1_ESM.pptx]

## Slide 1
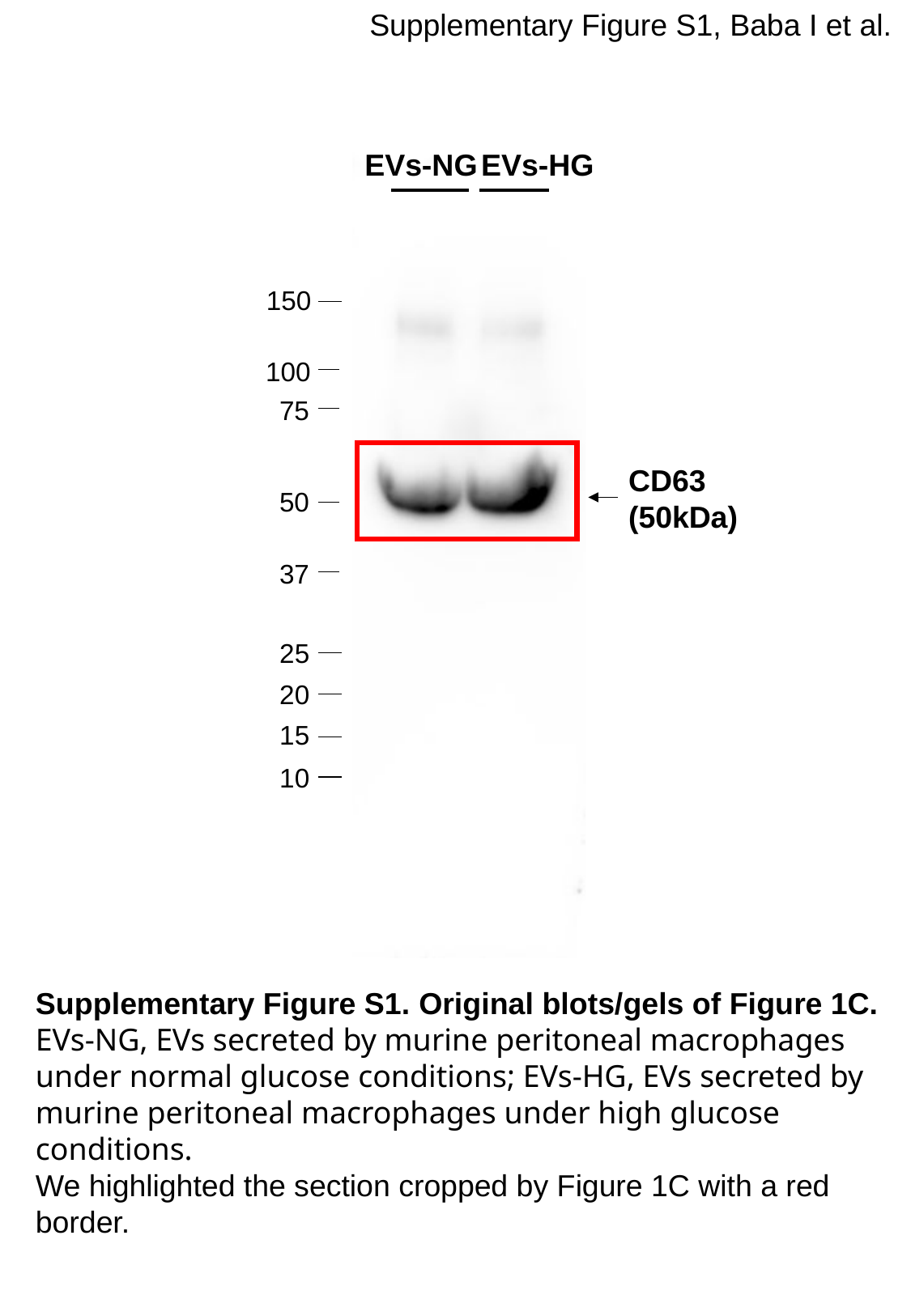

Supplementary Figure S1, Baba I et al.
EVs-NG
EVs-HG
150
100
75
CD63 (50kDa)
50
37
25
20
15
10
Supplementary Figure S1. Original blots/gels of Figure 1C. EVs-NG, EVs secreted by murine peritoneal macrophages under normal glucose conditions; EVs-HG, EVs secreted by murine peritoneal macrophages under high glucose conditions.
We highlighted the section cropped by Figure 1C with a red border.

## Slide 2
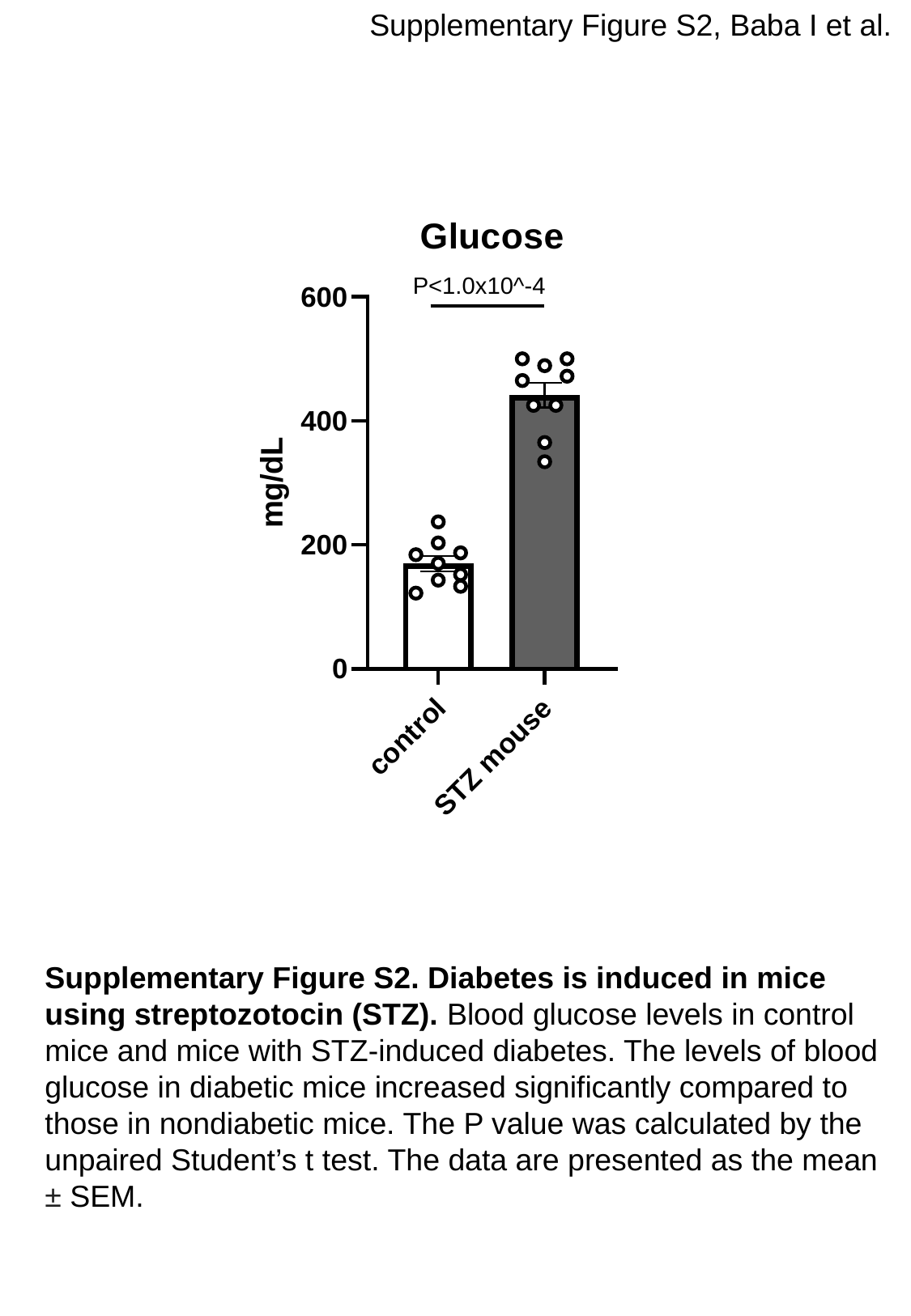

Supplementary Figure S2, Baba I et al.
P<1.0x10^-4
Supplementary Figure S2. Diabetes is induced in mice using streptozotocin (STZ). Blood glucose levels in control mice and mice with STZ-induced diabetes. The levels of blood glucose in diabetic mice increased significantly compared to those in nondiabetic mice. The P value was calculated by the unpaired Student’s t test. The data are presented as the mean ± SEM.

## Slide 3
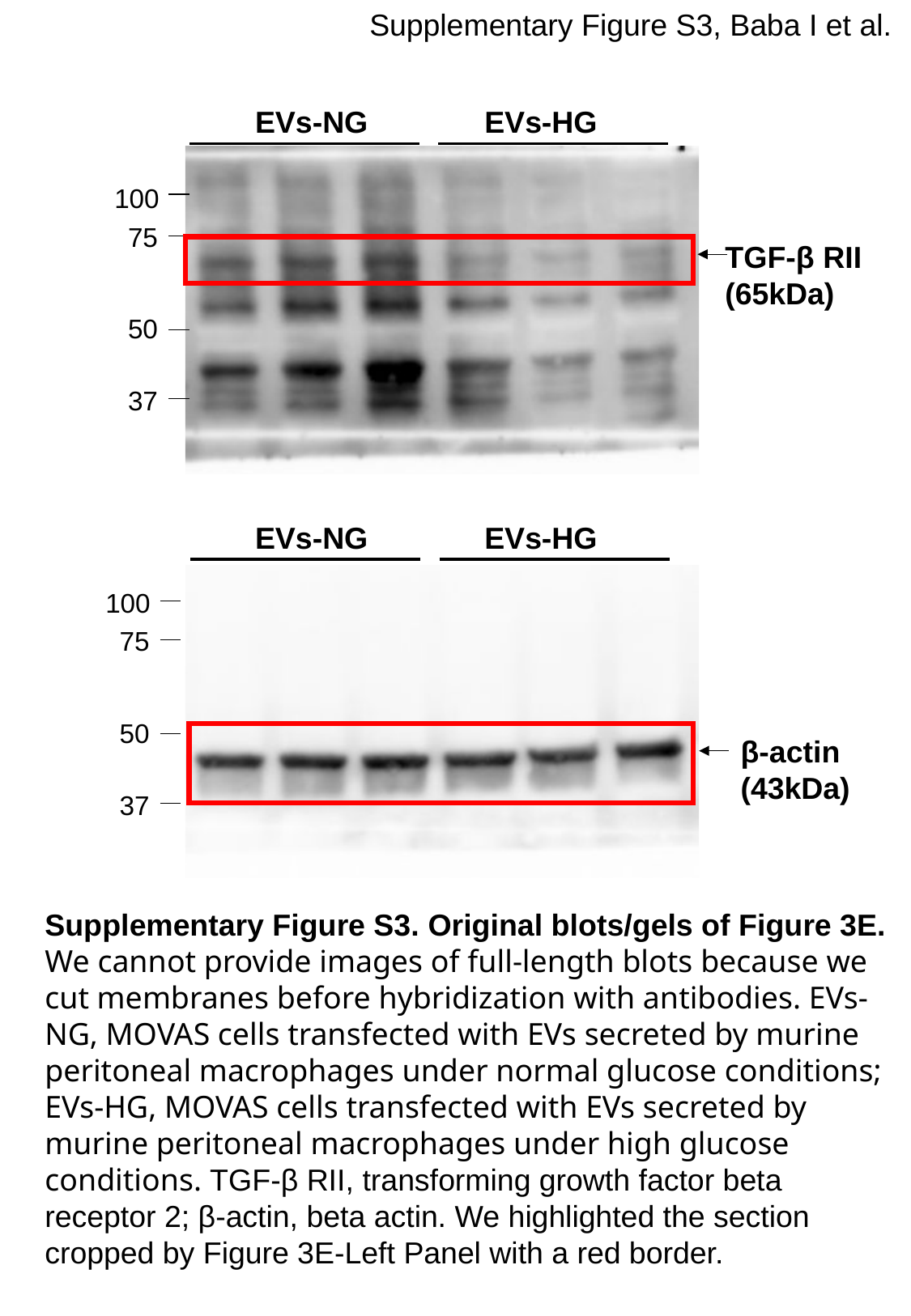

Supplementary Figure S3, Baba I et al.
EVs-NG
EVs-HG
100
75
TGF-β RII
(65kDa)
50
37
EVs-NG
EVs-HG
100
75
50
β-actin (43kDa)
37
Supplementary Figure S3. Original blots/gels of Figure 3E. We cannot provide images of full-length blots because we cut membranes before hybridization with antibodies. EVs-NG, MOVAS cells transfected with EVs secreted by murine peritoneal macrophages under normal glucose conditions; EVs-HG, MOVAS cells transfected with EVs secreted by murine peritoneal macrophages under high glucose conditions. TGF-β RII, transforming growth factor beta receptor 2; β-actin, beta actin. We highlighted the section cropped by Figure 3E-Left Panel with a red border.

## Slide 4
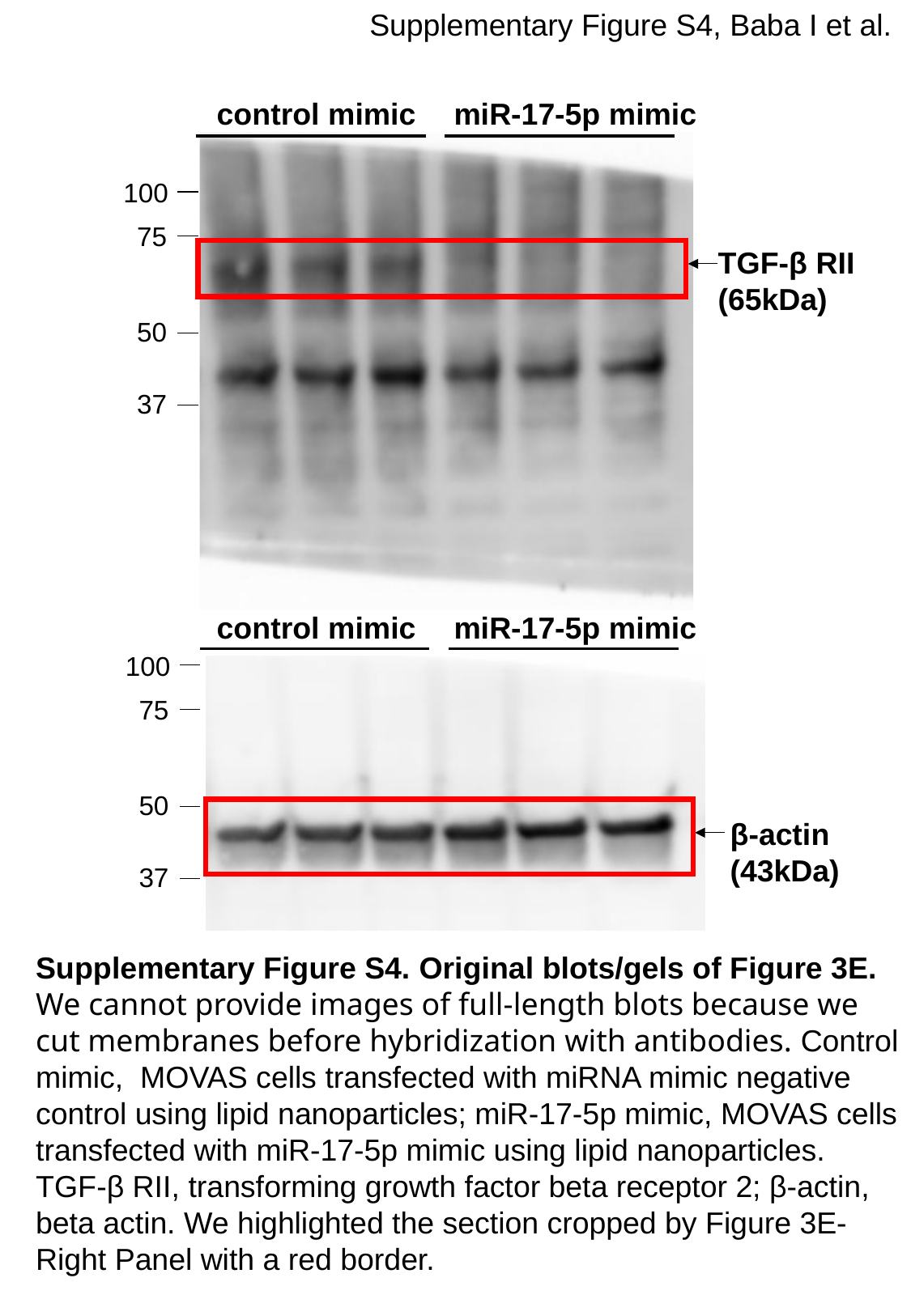

Supplementary Figure S4, Baba I et al.
control mimic
miR-17-5p mimic
100
75
TGF-β RII
(65kDa)
50
37
control mimic
miR-17-5p mimic
100
75
50
β-actin (43kDa)
37
Supplementary Figure S4. Original blots/gels of Figure 3E. We cannot provide images of full-length blots because we cut membranes before hybridization with antibodies. Control mimic, MOVAS cells transfected with miRNA mimic negative control using lipid nanoparticles; miR-17-5p mimic, MOVAS cells transfected with miR-17-5p mimic using lipid nanoparticles. TGF-β RII, transforming growth factor beta receptor 2; β-actin, beta actin. We highlighted the section cropped by Figure 3E-Right Panel with a red border.

## Slide 5
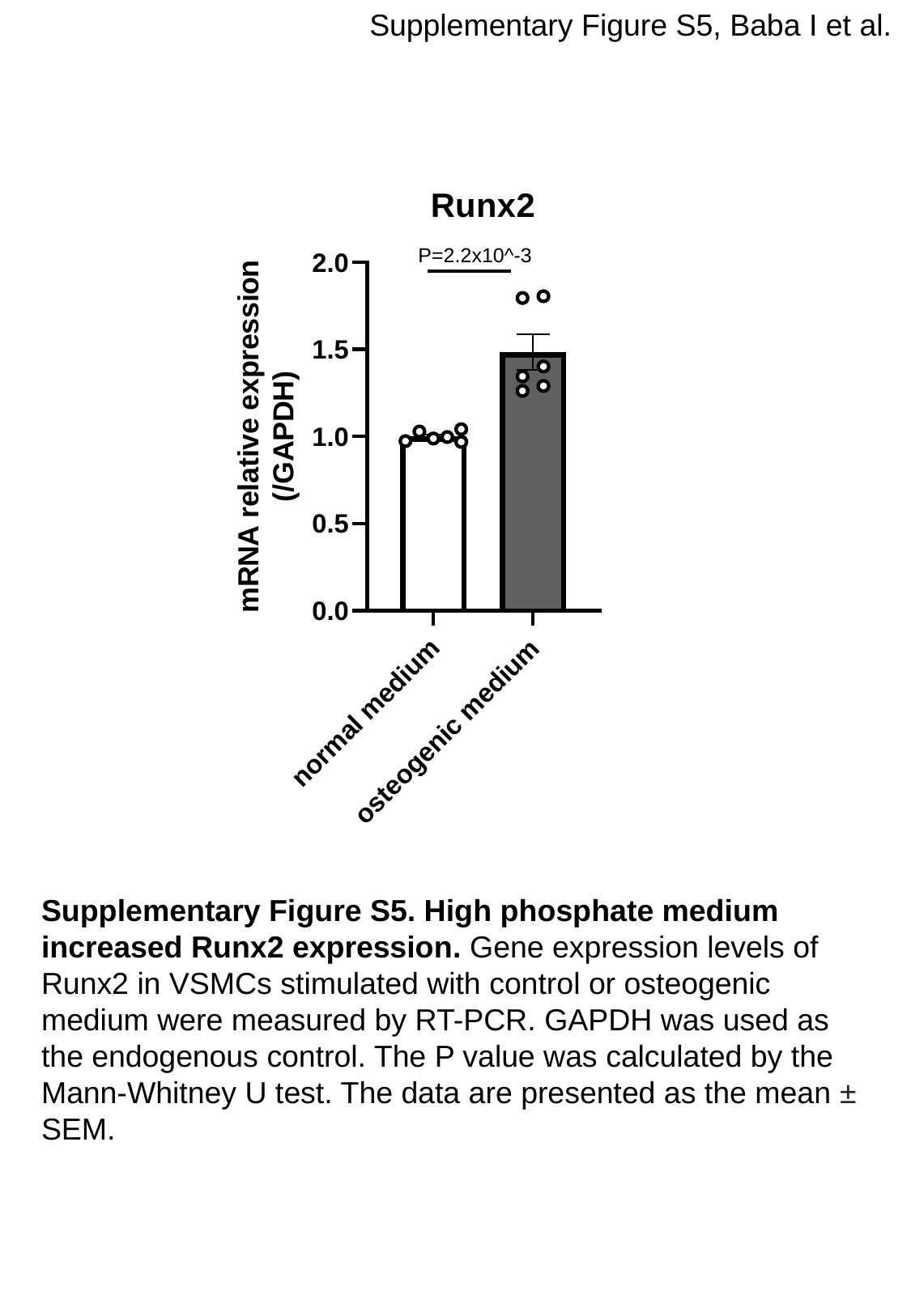

Supplementary Figure S5, Baba I et al.
P=2.2x10^-3
Supplementary Figure S5. High phosphate medium increased Runx2 expression. Gene expression levels of Runx2 in VSMCs stimulated with control or osteogenic medium were measured by RT-PCR. GAPDH was used as the endogenous control. The P value was calculated by the Mann-Whitney U test. The data are presented as the mean ± SEM.
