# Supplementary material for: EVs-miR-17-5p attenuates the osteogenic differentiation of vascular smooth muscle cells potentially via inhibition of TGF-β signaling under high glucose conditions
Source: Sci Rep. 2024 Jul 15;14:16323. doi: 10.1038/s41598-024-67006-9 (PMC11251274; doi:10.1038/s41598-024-67006-9)
Supplement: Supplementary file 2 — Supplementary Table 1. [file 41598_2024_67006_MOESM2_ESM.pptx]

## Slide 1
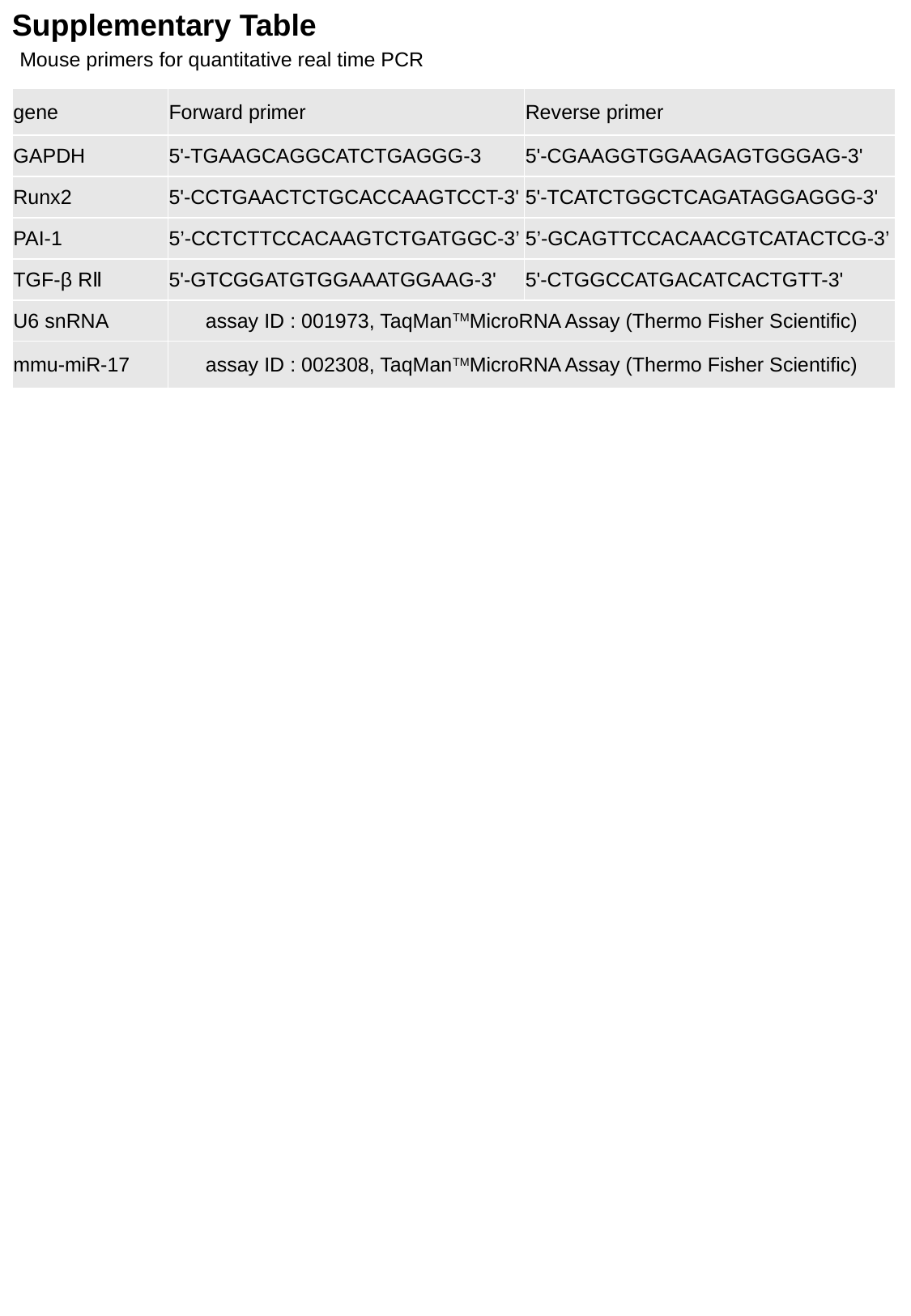

Supplementary Table
Mouse primers for quantitative real time PCR
| gene | Forward primer | Reverse primer |
| --- | --- | --- |
| GAPDH | 5'-TGAAGCAGGCATCTGAGGG-3 | 5'-CGAAGGTGGAAGAGTGGGAG-3' |
| Runx2 | 5'-CCTGAACTCTGCACCAAGTCCT-3' | 5'-TCATCTGGCTCAGATAGGAGGG-3' |
| PAI-1 | 5’-CCTCTTCCACAAGTCTGATGGC-3’ | 5’-GCAGTTCCACAACGTCATACTCG-3’ |
| TGF-β RⅡ | 5'-GTCGGATGTGGAAATGGAAG-3' | 5'-CTGGCCATGACATCACTGTT-3' |
| U6 snRNA | assay ID : 001973, TaqManTMMicroRNA Assay (Thermo Fisher Scientific) | |
| mmu-miR-17 | assay ID : 002308, TaqManTMMicroRNA Assay (Thermo Fisher Scientific) | |
